# Delayed Bacterial Neutrophil Recruitment and Bacterial Bone Dispersion: New Identified Factors in Peri‐Prosthetic Joint Infection Development. Insights From an Adult Minipig Model

**DOI:** 10.1111/apm.70031

**Published:** 2025-06-05

**Authors:** Katrine Top Hartmann, Anton Alexander Nolte Peterlin, Marie Høy Hansen, Julie Knippel Melsted Birch, Anders Odgaard, Bent Aalbæk, Mads Holm Christensen, Ida Thaarup, Thomas Bjarnsholt, Karen L. de Mesy Bentley, Andreas Petersen, Henrik Elvang Jensen, Louise Kruse Jensen

**Affiliations:** ^1^ Department of Veterinary and Animal Sciences University of Copenhagen Frederiksberg C Denmark; ^2^ Department of Orthopedics Herlev and Gentofte Hospital, University of Copenhagen Herlev Denmark; ^3^ Department of Orthopedic Surgery Copenhagen University Hospital, Rigshospitalet Copenhagen Denmark; ^4^ Department of Immunology and Microbiology, Costerton Biofilm Center University of Copenhagen Copenhagen Denmark; ^5^ Department of Clinical Microbiology Copenhagen University Hospital, Rigshospitalet Copenhagen Denmark; ^6^ Electron Microscope Shared Resource in the Center for Advanced Research Technology, Pathology & Laboratory Medicine University of Rochester Medical Center Rochester New York USA; ^7^ Statens Serum Institut Copenhagen S Denmark

**Keywords:** animal model, biofilm, implant infection, neutrophils, peri‐prosthetic joint infection

## Abstract

Clinically relevant animal models of peri‐prosthetic joint infection (PJI) are essential for studying infection initiation and progression. This study developed a PJI model in adult Göttingen minipigs, explicitly focusing on the early stages of infection to gain new perceptions of PJI initiation. The model was established by drilling a hole into the femoral head, followed by inoculation with either *Staphylococcus aureus* (*n* = 6) or saline (*n* = 4) and inserting a stainless‐steel screw. The animals were euthanized within 2 or 3 days post‐inoculation. Comprehensive bone and joint pathology analyses were performed. All *S. aureus* inoculated animals had bacteria reisolated from bone, screw, synovial fluid, and synovial membrane. Histology revealed numerous bacterial colonies in the peri‐implant bone tissue, many of which were unaccompanied by neutrophils, indicating delayed neutrophil recruitment to bacteria. In contrast, all synovial membrane‐located bacteria were recognized by the immune system. Digital pathology measures showed deep bacterial dispersion within the bone, at a far distance from the point of inoculation. This study presents a new PJI model, which facilitates the investigation of infection initiation and supports studies aimed at preventing PJI. The study uncovered two previously unknown insights into the development of PJI: delayed bacterial neutrophil recruitment and widespread osseous bacterial dissemination within 48 h.

## 1 Introduction

Peri‐prosthetic joint infection (PJI) following total joint replacement is a serious complication that can lead to implant loosening, pain, tissue necrosis, and functional impairment of the affected limb [1, 2]. The risk of developing PJI following total hip and knee replacement is estimated to be 1%–2% [3, 4, 5]. Although these percentages might seem low, they represent a substantial number of affected patients [6], and a revision for infection furthermore increases the risk for subsequent downstream revisions [7]. For example, the US project an annual total of up to 4 million primary hip and knee arthroplasties by 2030 [6]. Given that PJIs are recalcitrant hard‐to‐treat infections due to biofilm formation, they pose a severe healthcare burden, particularly also in the context of increasing antimicrobial resistance [1, 2]. To address these challenges, it is crucial to have clinically relevant animal models that allow for the study of PJI initiation and progression. However, the vast majority of existing PJI models focus solely on chronicity, aiming to prolong the infection duration for as long as possible. Furthermore, many of these models utilize small animal species such as mice, rats, and rabbits [3, 8]. Using small animals for PJI modeling presents challenges due to their size, which complicates the application of relevant humane orthopedic surgical procedures and multiple tissue sampling for different analyses [9, 10]. In contrast, pigs offer several advantages for studying orthopedic infections due to their bone anatomy, pathophysiology, and immune system, which all are highly comparable to humans [11, 12, 13]. While pigs often have been displaced as experimental subjects because of their rapid growth and large adult size, these challenges can be addressed with experimental minipigs, presenting outgrown adult pigs of a size that are feasible to handle [14].

Chronicity has been a central concern in PJI research since the first clinical observations of biofilms in 1978 [15] described how bacteria behave on foreign medical devices [16, 17]. Research into PJI chronicity addresses several critical questions: Why does PJI often develop months or even years after primary surgery? What causes PJI to progress slowly and to be nonresponsive to antibiotic treatment? Although these questions are fundamental, the earliest stages of PJI development have often been overlooked in efforts to investigate chronicity. Therefore, the focus herein is solely on the initial days after bacterial entry, which in most clinical cases occurs due to contamination at primary surgery or as a result of a later hematogenous spread [2].

The aim of the present study was to develop and validate a novel PJI model in adult Göttingen minipigs concentrating on the early stages of infection. The hypothesis was that a comprehensive investigation of the initial local bone and joint pathology in aged animals could facilitate identification of new perceptions in PJI development. The study provided two new insights or conceptualizations into the early development of PJI, which can help to explain the reasons behind the chronic nature of the clinical cases. The two new insights were delayed neutrophil recruitment and bacterial bone dispersion.

## 2 Methods

### 2.1 Animal and Housing

**FIGURE 1:**
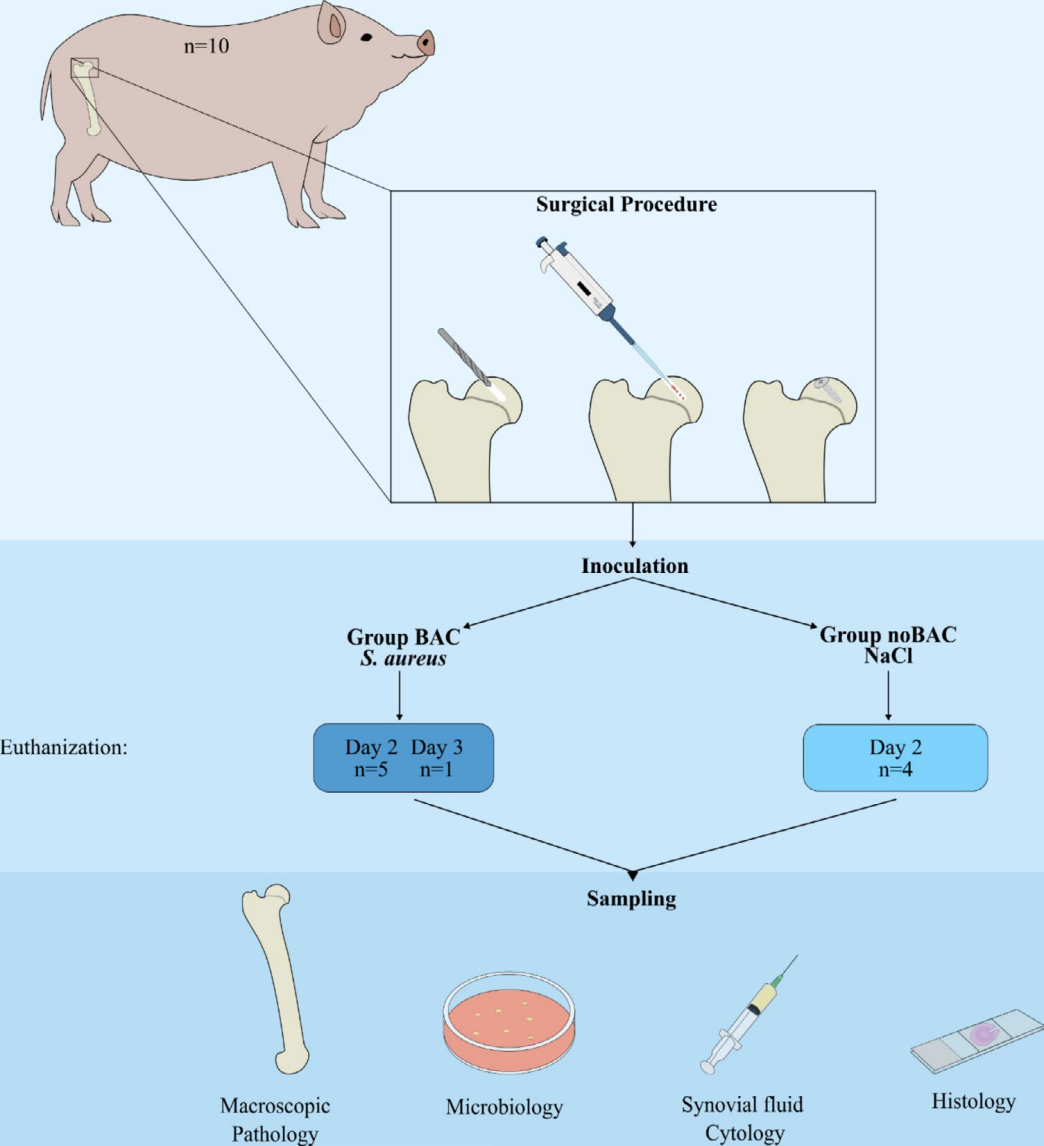
Study overview. Teen minipigs underwent hip surgery with insertion of a stainless‐steel screw into the joint surface of the femoral head of the hip joint. The minipigs either received *Staphylococcus aureus* or sterile saline as inoculum, followed by the insertion of the metal screw. Following euthanization on day 2 (*n* = 9) or day 3 (*n* = 1) sampling and evaluation were conducted.

The study included 10 adult female minipigs (Ellegaard Göttingen Minipigs A/S, Soroe Landevej 302, DK‐4261 Dalmose, Denmark), aged 23–27 months, with an average weight of 43 kg. The study design is outlined in Figure 1. Prior to the study, the minipigs had been used for breeding at Ellegaard Göttingen Minipigs and had 1–3 litters each. Two weeks of acclimatization were allowed prior to study start. The minipigs were housed in a barrier stable, in separate pens, with a 12‐h light/dark cycle. Authorized personnel inspected the animals several times a day, and daily clinical evaluations were conducted. The experiment was approved by The Danish Animal Experiments Inspectorate (license no. 2022‐15‐0201‐01130).

### 2.2 Anesthesia, Analgesia and Euthanization Protocol

A complete and detailed protocol (including doses and supplier) for analgesia, anesthesia, and euthanasia has recently been published [18]. In brief, the minipigs were sedated by an intramuscular injection of Zoletil Mixture. Anesthesia was maintained using intravenous Propofol, and operative analgesia by intravenous infusion of Fentanyl. An epidural block (between L6‐S1), consisting of Morfin, Bupivacain, and isotonic saline, provided operative and postoperative analgesia. Prior to surgery, the minipigs received an intramuscular injection of Meloxicam for postoperative analgesia, followed by oral Meloxicam once a day until euthanization. In case of increasing lameness or observed pain behavior, an intramuscular injection of Buprenorphine was provided every eighth hour. The minipigs were euthanized by an intravenous overdose of pentobarbital.

### 2.3 Surgical Procedure and Postoperative Care

Anesthetized minipigs were placed in left lateral recumbency, and the right lateral thigh was clipped and aseptically prepared as previously described [18]. The right hip joint was accessed using the anterior approach [18]. Once the femoral head was exposed, an implant cavity was created with a 2 mm drill, penetrating to a depth of 15 mm. Ten μL of inoculum (either 10^4^ CFU *S. aureus* or sterile saline) was injected into the prepared implant cavity. Immediately after inoculation, a stainless‐steel cancellous bone screw (Ø 3 mm, L 12 mm, head Ø 5 mm) was placed with subchondral fixation, avoiding bicortical penetration. This ensured that the screw head was counter‐sunk and did not protrude above the level of the joint cartilage. The joint capsule, muscle layers, subcutis, and cutis were closed consecutively. An antibiotic ointment was applied to the surgical wound (Fucidin 2% Ointment, Leo Pharma, Ballerup, Denmark), followed by Wound Plast spray (Kruuse, Langeskov, Denmark), to avoid environmental bacterial contamination. The minipigs were observed during their recovery period until they regained the ability to walk, eat, and drink. All animals underwent multiple daily inspections, which included assessments of wounds, walking ability, and overall well‐being. Impaired ability to stand, anorexia, and systemic signs of sepsis were set as humane endpoints.

### 2.4 Inoculum

*Staphylococcus aureus* S54F9 *spa*‐type t1333 was used for inoculation in six pigs (Group BAC) [19, 20, 21, 22]. The strain has been whole‐genome sequenced [23], is capable of producing biofilm [24] and is known to be highly virulent with genes encoding several toxins [23]. An inoculation dose of 10^4^ CFU in 10 μL of planktonic bacteria was chosen based on previous dose–response studies conducted in a porcine implant‐associated osteomyelitis model [19]. Four control animals (Group noBAC) were inoculated with 10 μL of sterile isotonic saline.

### 2.5 Radiographs

During sedation prior to euthanasia, radiographs of the right hip joint were obtained to evaluate the correct screw position. The radiographs were obtained in lateral and ventro‐dorsal positions using an X‐ray system (Shimadzu Radspeed MC, Fuji, Tokyo, Japan) set to 79 kV and 18 mAs.

### 2.6 Sampling and Macroscopic Pathology

After euthanasia (2‐ or 3‐days post‐surgery), the surgical wound was inspected and opened in layers down to the hip joint, using sterile instruments. Microbiology samples were collected from the incision line, the subcutaneous sutures, the inflicted muscles, and the joint capsule. The joint capsule was opened aseptically, and synovial fluid samples were collected. Besides microbiology analyses, the synovial fluid was evaluated macroscopically for color, turbidity, and viscosity, and smears were made for cytology including differential cell counts. The synovial membrane was inspected for signs of inflammation such as hyperemia and hyperplasia and collected for microbiology and histology. The entire femoral bone was removed, and the screw was aseptically sampled for sonication. Following this, a central sagittal section was made through the femoral screw cavity. The bone was evaluated for early lesions including hematoma, pus, and necrosis. Additionally, samples for histological and microbiological analysis were collected from the bone‐screw interface. The left hip joint and femur were sectioned and sampled correspondingly to the right. The left and right major deep inguinal lymph nodes were collected for histology. All abdominal and thoracic organs were inspected in situ. Samples were collected for histology from the left caudal lung lobe, liver, and right kidney. Additionally, a sample from the right caudal lung lobe was collected for microbiology.

### 2.7 Microbiology

Before inoculation and before euthanasia, venous blood samples were collected for microbiological assessment. All blood and tissue samples were prepared and cultured as recently described [18]. Quantitative microbiology was performed for bone tissue and screws only. Following the removal of screws, they were placed in cryo tubes, covered with 1 mL sterile 0.9% NaCl, and kept on ice until sonication. The tubes were sonicated in an ultrasound bath, followed by quantitative microbiology as previously described [25], except culturing was performed on Lysogeny Broth (LB) agar plates and incubated for 24 h. *Spa‐typing* of all isolates of *S. aureus* from bone and implants was performed [26]. All microbiological evaluations were performed blinded to group allocation.

### 2.8 Histology and Immunohistochemistry

All soft tissue samples were fixed in 10% buffered formalin for 5 days. Bone samples were fixed in formalin under alternating cycles of 2 h of vacuum (−0.1 MPa) followed by 2 h of normal atmospheric pressure for 48 h before decalcification in ethylene‐diaminetetraacetic acid (EDTA) for 4 weeks. After fixation and decalcification, all samples were trimmed and processed through graded concentrations of alcohol and xylene, embedded in paraffin wax, and sectioned to a thickness of 4–5 μm. All sections were stained with Hematoxylin & Eosin (HE). Additionally, bone samples were also stained with PTAH for the identification of fibrin and immunohistochemically (IHC) with antibodies towards *Staphylococci* [24] as well as calprotectin, which is found in macrophages, monocytes, and granulocytes [27]. Synovial membranes were also subjected to IHC staining for *Staphylococci*. Detailed IHC protocols can be found in the references [20]. The implant cavity and the surrounding osseous tissue were assessed for debris, erythrocytes, inflammatory cells, bone marrow cells, fibrin, neutrophil traps (NETs), and bacteria. The number of infiltrating tissue neutrophils was counted and scored as previously described by Morgenstern [28]. Briefly, at least 10 400× magnification high‐power fields (HPF) in each section were examined, and the extent of inflammatory cells was scored into one of four categories (0, 1, 2, 3): absence of neutrophils (NPs); an average of less than one NP/HPF; an average of one to five NPs/HPF; and an average of more than five NPs/HPF.

Positive *S. aureus* IHC‐stained bacterial colonies were counted, with counting ceasing at 100 colonies. Similarly, for the synovial membranes, neutrophils and IHC‐positive *S. aureus* bacteria were quantified and the presence of NETs evaluated. All bone sections were scanned to digital slides using a Zeiss Axioscan 7 microscope slide scanner (Zeiss, Oberkochen, Germany) with a 20×/0.8 objective. Digital scans were analyzed using Qupath software version 0.4.4 [29]. The line‐annotation tool was used to measure the distance between the implant cavity (point of inoculation) and osseous tissue containing IHC‐positive *S. aureus* bacteria located farthest away. The wand‐annotation tool was used to identify and measure IHC‐positive *S. aureus* colonies larger than 30 μm^2^.

### 2.9 Historic Controls

The early inflammatory bone response of the adult PJI minipigs was compared to findings from a porcine hematogenous osteomyelitis model [30] based on juvenile 8‐weeks‐old female York‐Landrace crossbreed pigs. The hematogenous model was inoculated with the same bacterial strain as used in the present study and euthanized at comparable time points, i.e., 24 (*n* = 4) and 48 (*n* = 4) hours following intravenous inoculation. Stored paraffin‐embedded femoral bone lesions from the hematogenous model were stained with HE and IHC for *Staphylococci* and evaluated with regard to neutrophil response and bacterial localization.

### 2.10 Transmission Electron Microscopy

Selected histological bone sections from two infected minipigs euthanized 2‐ and 3‐days post infection were evaluated with electron microscopy to assess bacterial activity and whether the bacteria had entered the osteocyte lacuna‐canaliculi network (OLCN). The sections were prepared by the Paraffin Section Pop‐off Technique, which has previously been described in detail [31]. In brief, the glass slides labeled by immunocytochemistry were examined by light microscopy to determine the region of interest (ROI) for the “pop‐off” technique [31]. The ROI was marked on the backside of the slides using circular etching and a diamond pen. The coverslips were removed in xylene, and the slides were rehydrated back to water, then fixed overnight in 2.5% phosphate buffered glutaraldehyde at 4°C. The slides were then rinsed and incubated in phosphate buffered 1.0% osmium tetroxide for 20 min, rinsed in 2 changes of distilled water, dehydrated through a graded series of ethanol to 100% (×3), infiltrated with Spurr's epoxy resin (1:1100%/Spurr for 60 min), then 100% Spurr's overnight. The next day, several size 3 BEEM capsules were filled with fresh resin, inverted, and placed over the ROI (that had been etched on the back side of the glass slides) then placed into a 60°C oven to polymerize overnight. The polymerized BEEM capsules are “popped off” the slides by dipping several times into liquid nitrogen and then gently wiggling them to break the surface tension between the glass surface and the entrapped tissue section within the polymerized epoxy capsule. The capsules were trimmed to the ROI with a razor blade to a small trapezoid face for thin sectioning at 70 nm thinness using a Leica UC7 ultramicrotome and a diamond knife. The thin sections were placed onto nickel carbon coated slot grids and examined using a Hitachi 7650 Transmission Electron Microscope with image capture using an attached Gatan Erlangshen 11‐megapixel digital camera.

### 2.11 Statistics

Statistics were performed in GraphPad Prism version 10.2.3 for Windows. Comparisons of neutrophil counts in bone and synovial membrane between Groups BAC and noBAC were analyzed by unpaired *t*‐tests, while comparisons within the two groups were analyzed with paired t‐tests. *p*‐values ≤ 0.05 were considered statistically significant.

## 3 Results

### 3.1 Clinical Observations and Screw Position

**FIGURE 2:**
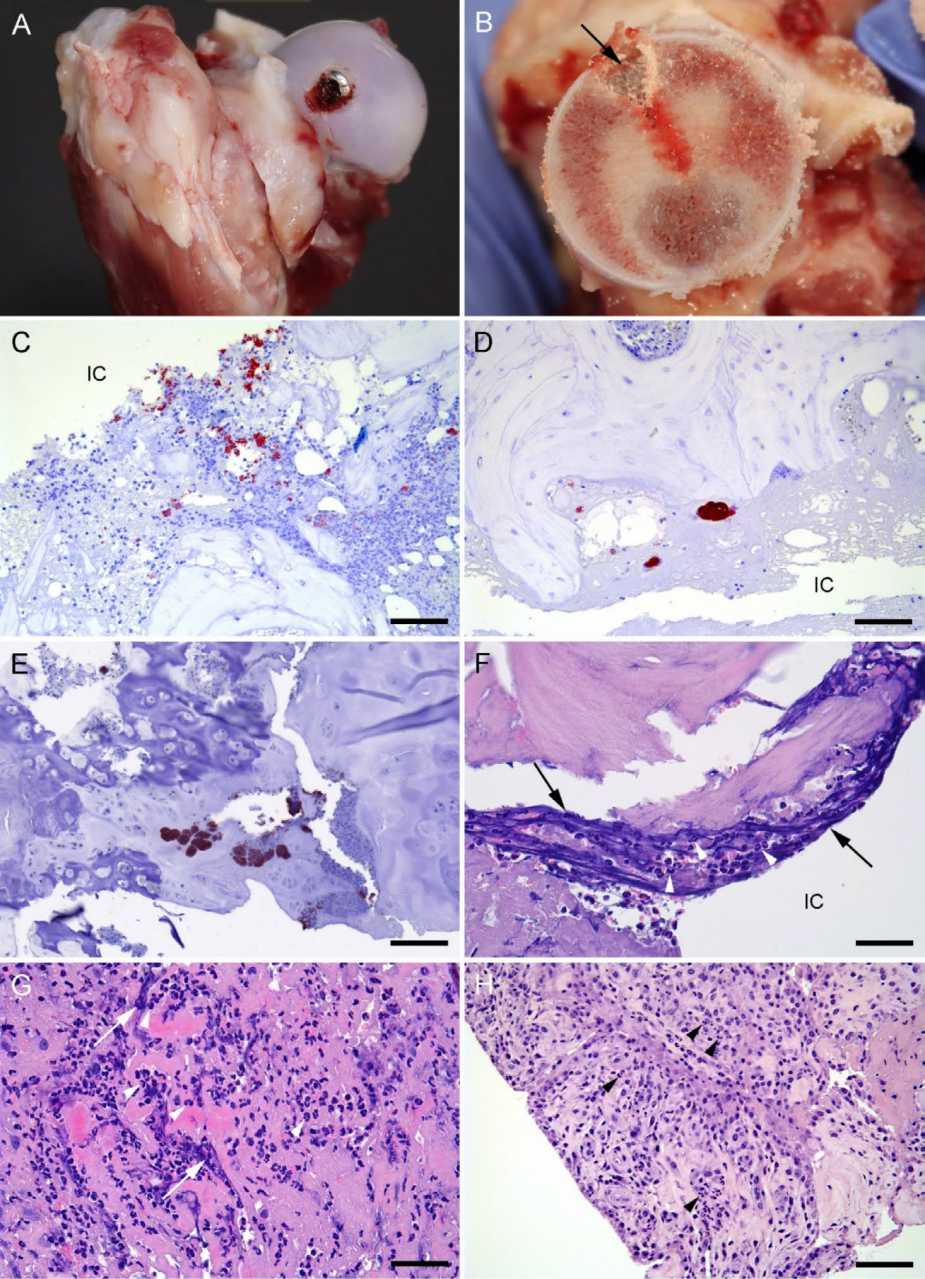
Presentation of gross and histological findings in the bones and joints in a peri‐prosthetic joint infection minipig model. (A) The screw inserted into the femoral head. (B) Sagittal section of the femoral bone through the implant cavity (arrow). Slight hyperemia is seen in the borders of the implant cavity. (C) Positive *Staphylococcus aureus* (red‐brown) colonies accompanied by neutrophils in the necrotic debris at the border of the implant cavity (IC), from a minipig euthanized 2 days after inoculation of *S. aureus* . IHC, bar = 100 μm. (D) Positive (red‐brown) *S. aureus* colonies unaccompanied by neutrophils, at the border of the implant cavity (IC), IHC, bar = 70 μm, and (E) in the cartilage of the remnant physis line, IHC, bar = 100 μm. From a minipig inoculated with *S. aureus* and euthanized 2 days after inoculation. (F) Neutrophils (arrowheads) at the border of the implant cavity (IC) at some necrotic bone debris, the basophilic threads (arrows) surrounding the neutrophils are neutrophil traps (NETs). From a minipig euthanized 2 days after inoculation of *S. aureus* . HE, bar = 75 μm. (G) Synovial membrane from a minipig euthanized 2 days after inoculation of *S. aureus* . A high amount of neutrophils (arrowheads) are present in the tissue, surrounded by NETs (arrows) seen as basophilic threads. HE, bar = 75 μm. (H) Synovial membrane from a minipig euthanized 2 days after inoculation with saline. An increased number of neutrophils are present in the tissue (arrowheads), however not as marked as in (G), and without any NETs. HE, bar = 150 μm.

After recovering from anesthesia, all 10 minipigs exhibited slight lameness in the operated leg, i.e., they could still walk freely around their pens, eat, and drink. The slight lameness of Group BAC minipigs worsened to severe lameness at 48 h after inoculation. The minipigs of Group noBAC also showed an initial temporary slight lameness, which did not require any additional analgesic treatment. Radiographic examination and postmortem evaluation confirmed that all screws were correctly inserted into the femoral head (Figure 2A).

### 3.2 Macroscopic Pathology Findings

Hemorrhage/hematoma and edema were observed in subcutis and musculature adjacent to the incision line of all animals. The gross lesions observed in Group BAC animals were bone hyperemia (Figure 2B) and right‐side synovial membrane hyperemia and hyperplasia. Hyperemia of the synovial membrane was only observed in two Group noBAC animals.

### 3.3 Microbiology

All blood samples, lung samples, and left‐side synovial fluid samples were sterile, indicating no systemic bacterial spread. In all Group BAC animals, *S. aureus* spa‐type t1333 (inoculation strain) was re‐isolated from the right‐side synovial membrane, synovial fluid, bone tissue, and screws. The mean CFU numbers of screws and bone tissue were 1.3 × 10^6^ CFU/mL and 1.1 × 10^6^ CFU/g, respectively. *Staphylococcus aureus* was also re‐isolated from subcutaneous and musculature sutures in two and four Group BAC animals, respectively. However, *S. aureus* was not found in any cutis samples from the incision lines. All samples from the right hind leg of Group noBAC animals were free of *S. aureus* .

### 3.4 Synovial Fluid

Group BAC animals showed > 80% neutrophils at the cytological examination of the synovial smears, whereas Group noBAC animals had below 80% neutrophils. Synovial fluid from the right hip joint of all animals contained a moderate number of erythrocytes and had a decreased viscosity. From the left hip joint, neutrophil counts varied from a few to 40% in all minipigs; the samples were sparse, of normal viscosity, and mixed with few to moderate numbers of erythrocytes.

### 3.5 Histology and Immunohistochemistry

No histopathological changes were found in the lung, liver, kidney, or lymph nodes of any animals. In all animals, the implant cavity was enclosed by a rim of necrotic trabecular bone intermingled with fibrin threads (confirmed by PTAH staining), erythrocytes, and a varying degree of inflammatory cells and bone marrow cells. In all Group BAC animals, a high number of IHC‐positive bacterial colonies (> 100) were identified in both the bone and the synovial membrane. Inside the bone, the bacterial colonies were located at the implant cavity interface and deeper within the surrounding bone tissue. In the synovial membrane, the colonies were found on the synoviocyte lining surface and within the fibrovascular tissue. Conversely, no bacterial colonies were present in Group noBAC.

**FIGURE 3:**
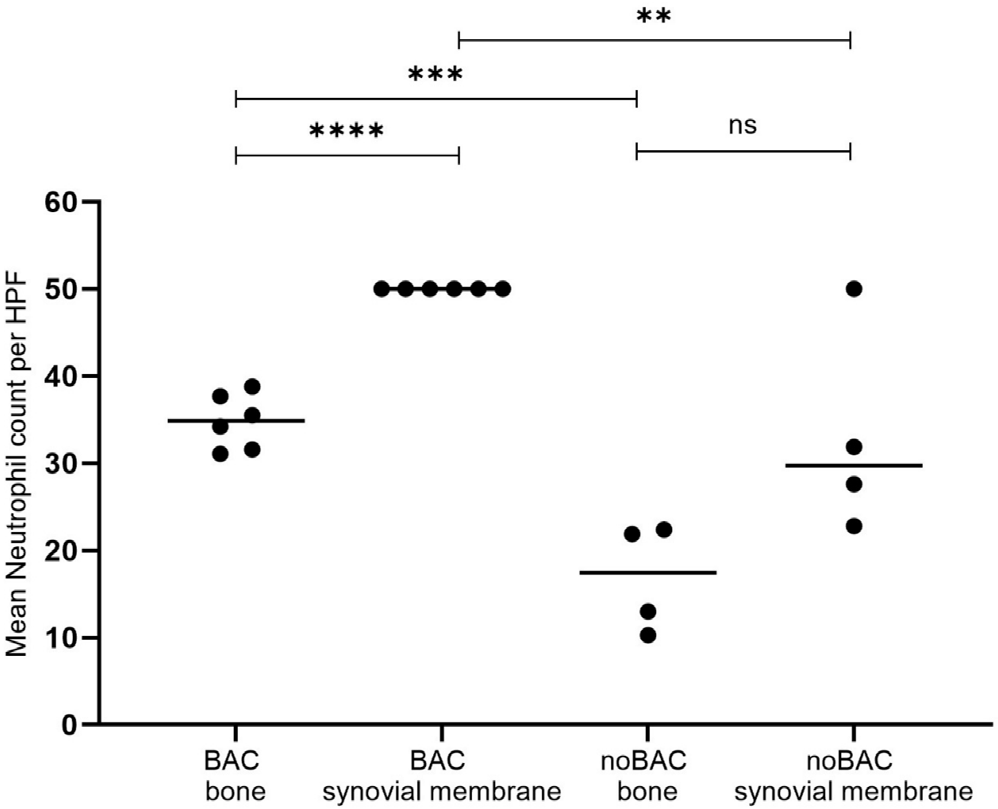
Histological scoring of neutrophils in the right femoral bone and in the synovial membrane from the right hip joint. The neutrophils were scored by counting all neutrophils up to 50 in 10 high power fields (HPFs) at 400× magnification, with the max mean neutrophil count per HPF being 50 neutrophils. The mean neutrophil count in both bone and synovial membranes were found significantly higher (*p* < 0.001 and *p* < 0.01 respectively) in the infected Group BAC than in the control Group noBAC. A significantly higher (*p* < 0.0001) neutrophil count was seen in the synovial membrane compared to bone tissue within the Group BAC, while no significance was seen within the Group noBAC.

Osseous and synovial membrane infiltrating neutrophils (confirmed by IHC positive calprotectin staining) were present in all Group BAC and noBAC animals, with a neutrophil score of 3 for both groups (Figure 2C). However, the mean neutrophil counts for both compartments were significantly higher for infected animals (Figure 3). Within Group BAC, a significantly higher neutrophil count was seen in the synovial membrane compared to bone tissue (Figure 3), while no significance was seen for Group noBAC (Figure 3). The neutrophil infiltration in Group BAC animals was accompanied by surrounding NETs in both bone and synovial membrane, whereas no NETs production was identified in any Group noBAC animals (Figure 2F–H).

**FIGURE 4:**
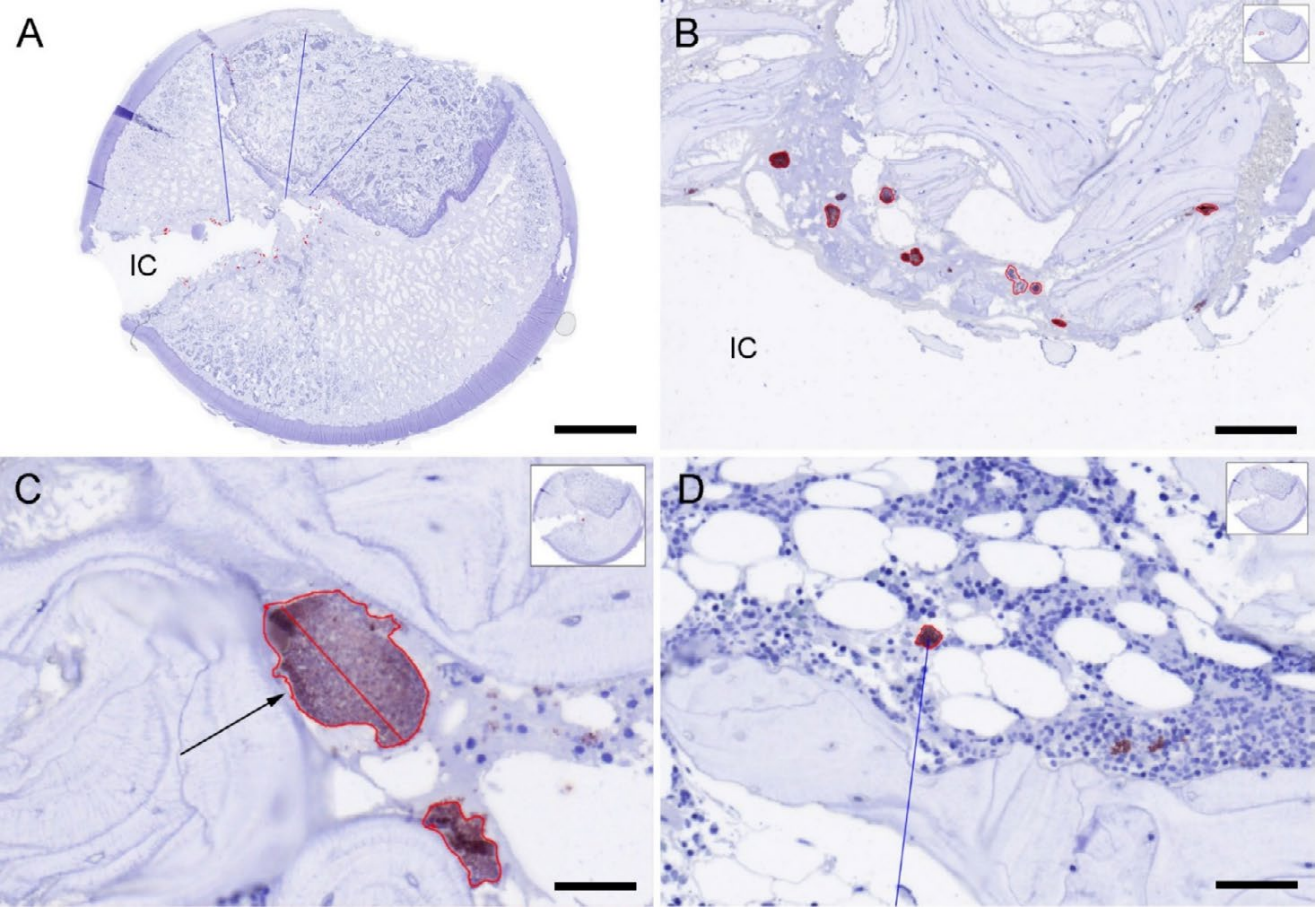
Presentation of the Qupath measurements in the histological bone sections in a peri‐prosthetic joint infection minipig model, from a minipig euthanized 2 days after inoculation of *Staphylococcus aureus* . (A) Histological overview of the femoral bone with the implant cavity (IC). Blue lines are the measurements of the distance between the implant cavity (IC) and the bacteria situated at the longest distance from the implant cavity (IC). While the red markings are bacterial colonies (larger than 30 μm^2^) which is unaccompanied by neutrophils. IHC, bar = 3,5 mm. (B) Positive (red‐brown) *S. aureus* colonies, larger than 30 μm^2^, unaccompanied by neutrophils, at the border of the implant cavity (IC), marked with red and measured by the Qupath annotation tool IHC, bar = 120 μm. (C) The largest *S. aureus* positive colony, unaccompanied by neutrophils, (arrow), the outline of the colony and the length through the center is marked, IHC, bar = 40 μm. (D) Close up of one of the three bacterial colonies situated at the longest distance from the implant cavity (IC), measured by the blue line, IHC, bar = 90 μm.

Examination of the distribution of infiltrating neutrophils in Group BAC identified that many of the cells were not associated with bacterial colonies. Notably, all infected animals had > 20 bacterial colonies in their bones (larger than 30 μm^2^) with no neutrophils in their proximity (Table 1, Figure 2D,E and Figure 4). Unaccompanied bacterial colonies were observed within necrotic debris of the interface, as well as in the adjacent bone marrow intermingling with trabecular bone, and within the cartilage of the remnant physis line. The largest unaccompanied colony measured 31,381 μm^2^ (Table 1 and Figure 4C). Conversely, bacterial colonies located in the synovial membrane were consistently surrounded by neutrophils. Furthermore, it was observed that the inoculated bacteria were dispersed into the marrow space of the surrounding bone (Figure 4A,D). In all cases, some of the inoculated bacteria were located away from the inoculation site deeper into the adjacent bone. The most distant osseous bacterial colony was found 7438 μm within the trabecular network (Table 1).

**TABLE 1:** Histological measurements of immunohistochemically (IHC) positive *S. aureus* colonies of the infected animals from Group BAC.

Group	Days in study	Distance (μm) from inoculation point to *S. aureus* farthest away	Mean distance (μm) from inoculation point to the three *S. aureus* colonies farthest away	*S. aureus* colonies larger than 30 μm^2^ undetected by neutrophils (cease counting at 20 colonies)	Size (μm^2^) of largest undetected colony	Length (μm) of the largest (by area μm^2^) undetected colony
BAC	2	5000	4937	> 20	333	86
BAC	2	1265	1140	> 20	693	146
BAC	2	2389	1914	> 20	1950	100
BAC	2	7438	7320	> 20	3138	79
BAC	2	1878	1351	> 20	1758	85
BAC	3	2535	2015	> 20	1176	62

Examination of historic femoral metaphyseal bone lesions from pigs with experimental hematogenous osteomyelitis [30] revealed a massive neutrophil infiltration corresponding to a score of 3 and a maximum accessible number of neutrophils, i.e., > 50 in all animals. All neutrophil infiltrations showed bacterial recognition and mobilization around bacterial colonies. Unaccompanied bacterial colonies were not identified in any of the historic animals.

### 3.6 Transmission Electron Microscopy

**FIGURE 5:**
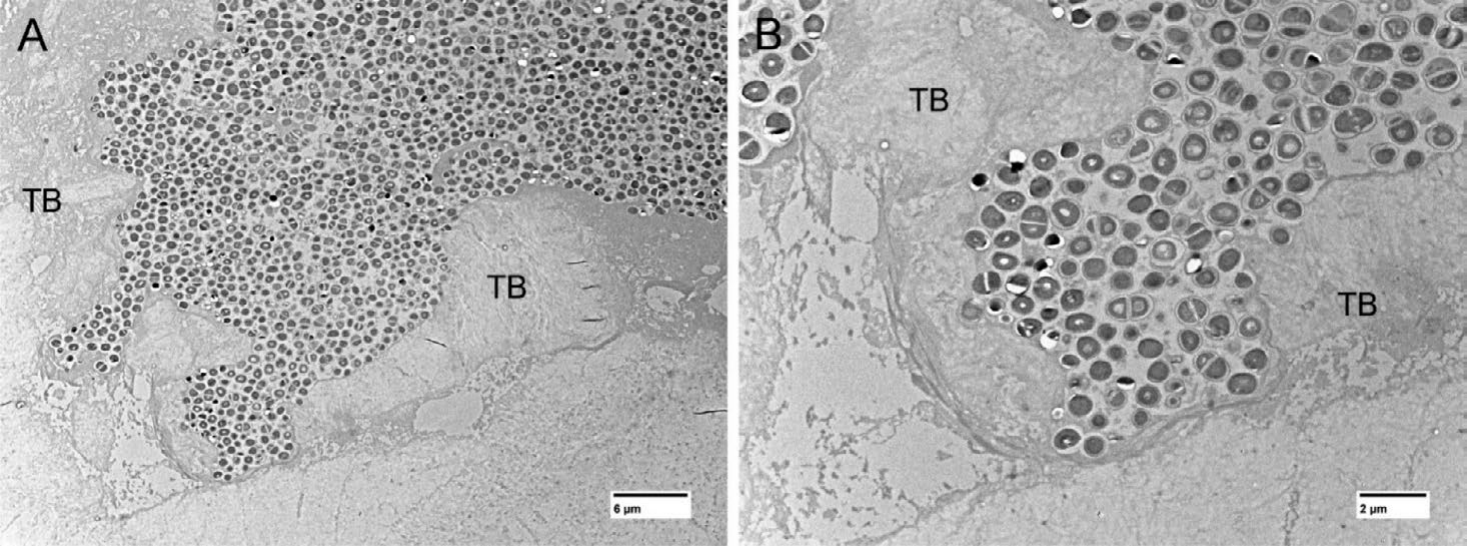
Transmission Electron Microscopy (TEM) images of the right femoral head from a minipig with experimentally induced prosthetic joint infection euthanized 2 days following inoculation. A vast amount of actively dividing bacterial cells were seen next to the trabeculae bone (TB) without the presence of neutrophils.

Evaluation by transmission electron microscopy (TEM) did not show bacterial invasion of the OLCN but did reveal a vast amount of actively dividing bacteria in the bone marrow next to trabeculae bone (Figure 5).

## 4 Discussion

The present minipig PJI model successfully replicates the most common pathogenesis of PJI i.e., intra‐operative infection, with a 100% infection rate. All infected animals fulfilled the EBJIS criteria for diagnosing PJI [32], in respect to both synovial fluid neutrophils, histology, and microbiological examination criteria. However, the histological scoring with ≥five neutrophils in ≥five HPF is only confirmative for infection in chronic cases [32], as elevated neutrophil counts also occur in response to surgery per se. Nonetheless, the infected minipigs still had a higher mean neutrophil count than the uninfected control group, indicating a clear increased recruitment in response to the inoculated bacteria.

However, despite the significantly increased recruitment of neutrophils, the vast majority of bacterial colonies inside the bones were unaccompanied and thus undiscovered by the immune cells. Simply put, most bacterial colonies, both at the screw‐bone interface and deeper into the bone marrow, were not surrounded by neutrophils and had no neutrophils in their proximity. This delayed osseous bacterial neutrophil recruitment represents an anomaly against the background provided by a central paradigm of pathology, namely that neutrophils are recruited from the bone marrow in response to tissue damage and pathogens by a consistent time pattern that starts and peaks after 2 and 24 h, respectively [33]. Specifically, the present study identified up to 72 h of delayed insufficient bacterial neutrophil recruitment to the peri‐implant tissue. Delayed neutrophil recruitment has also recently been reported in other implant‐related infection models. In a murine model based on a 24 h infected peritoneal silicone implant, a heterogeneous spatial neutrophil distribution was observed and pre‐grown surface‐attached bacterial aggregates remained undiscovered [34]. Another murine study based on intravital imaging of infected skin implants revealed that pre‐grown surface‐attached bacteria grew and remained undiscovered by neutrophils for up to 3 h [35]. The lack of infiltrating neutrophils in the Group BAC minipigs could be due to drilling and thermal‐induced damage, resulting in necrotic tissue and a disrupted local blood supply [36]. In contrast, a huge attraction of neutrophils was seen in the well‐vascularized synovial membrane. Another explanation for delayed bacterial neutrophil recruitment is a diminished chemo‐attractant signaling of the bacterial attendance to the immune system, which in the present study could be explained by tissue necrosis, animal age, and anatomical location. The present study applied fully grown adult minipigs, which adds to the relevance of the model, since PJI primarily is a problem concerning adults, especially the elderly and often in combination with immunodeficiency [5, 6, 36]. The femoral head and proximal femur of the applied adult minipigs showed, despite some adipocytes, an extremely low cellular content, i.e., hematopoietic cells and osteoblasts. Several studies of osteoimmunology have identified that osteoblasts express toll‐like receptors and acute phase proteins, which are involved in the initiation of the innate immune response [37, 38]. In comparison, the historic hematogenous osteomyelitis model, based on young pigs, showed a higher cellular bone density of the metaphysis, and delayed bacterial neutrophil recruitment was not observed [30]. Thus, a combination of aged individuals, anatomical areas of low cellularity and immune function, necrotic tissue i.e., disrupted local blood supply, and the presence of a foreign surface might cause a delayed bacterial neutrophil recruitment predisposing to PJI development.

*Staphylococcus aureus* is a major pathogen in PJI and, therefore, it was used as inoculum in the present study. It is tempting to speculate that *S. aureus* , and other pathogens as well, can benefit from delayed neutrophil recruitment, as the delay represents a critical time window in which the bacteria can initiate biofilm development and develop immune tolerance. In Group BAC minipigs, undiscovered bacterial aggregates had a mean size of app. 1.500 μm^2^. However, *S. aureus* aggregates have previously demonstrated neutrophil phagocytic tolerance and the ability to lyse neutrophils already at a size of 50 μm^2^ [34]. Furthermore, a high surface density of neutrophils was proven necessary for effective implant clearance of *S. aureus* [34]. The present 2D length (diameter) measurements of bacterial aggregates (from 62 to 146 μm) were performed to make comparisons to clinically identified biofilms [39]. A large review on in vivo biofilms identified that biofilms of orthopedic infections like PJI and chronic osteomyelitis had a diameter of app. 50 μm [39]. Therefore, based on the present study, contaminating *S. aureus* bacteria can within days establish the final biofilm size and from here, it is a question of biofilm maturation towards acquiring antibacterial tolerance and persister‐cell formation [39, 40, 41]. Although the bacterial aggregates of Group BAC minipigs had a size associated with tolerance to neutrophil clearance, they still showed cellular divisions (TEM pictures, Figure 5) which could indicate susceptibility to killing by many antibiotics.

The observed location of bacteria far from the inoculation site is highly surprising since *S. aureus* historically has been reported as non‐motile [42]. Motility by binary fission has been reported for *S. aureus* among others when invading the lacunae‐canaliculi system [42, 43]. But motility by binary fission does not alone seem to explain how the bacteria can be found so widely spread throughout the femoral head after 48 h. One explanation could be that during the insertion of the screw, the screw acts like a piston inserted into the fluid‐filled cavity, thereby replacing the volume of the fluid. This leads to a high pressure which forces the fluid away from the cavity and deeper into the bone [44]. Another explanation of the widespread bacteria within the bones could be osseous extracellular fluid flow. In bones subjected to load, e.g., during weight‐bearing, extracellular fluid flows through the macropores of the bone marrow as well as through the micropores of the lacunae‐canaliculi system and the Volkmann and Haversian canals [45]. Furthermore, load pressure gradients and velocity cause the bone marrow cells to move relative to each other [45]. Based on these numbers, it seems reasonable to suggest that bacterial bone flow occurred in the Group BAC minipigs. Furthermore, the load‐generated fluid flow highlights the importance of using functional implants, inserted at clinically relevant sites and subjected to load, if all aspects of infection initiation are to be considered in PJI animal models. Bone flow can potentially occur until the bacteria get trapped by an inflammatory response and, therefore, delayed discovery and neutrophil recruitment towards bacterial contamination represent a risk of increasing the infected area.

## 5 Conclusion

In PJI, the early interactions between the pathogen and host are unknown; therefore, to address this gap, a novel adult minipig PJI model was developed to investigate the early bone and joint pathology. The minipig PJI model demonstrated 100% reproducibility, i.e., all inoculated animals developed an infection alongside high reliability as the interplay between neutrophils and bacteria could be investigated in adult species with a comparable immune system to humans [46]. The model was designed for high throughput, allowing researchers to produce multiple individuals while minimizing animal suffering compared to full‐scale hip replacement. The model has the potential to be applied in studies of preventive strategies, i.e., antimicrobial coatings, gels, or cements, as well as for the investigation of diagnostic tools. Specifically, the adult minipig PJI model revealed several important findings: (1) there is a delay in the recruitment of neutrophils to bacteria following surgical contamination; (2) during this delay, the bacteria can reach a mature biofilm size and a size that is associated with neutrophil tolerance; and (3) the contaminating bacteria can get widely dispersed inside the bone until trapped by inflammation. These newly characterized initial events may help explain the clinical situation of slow‐onset infections and the development of chronicity seen in PJI.

## Conflicts of Interest

The authors declare no conflicts of interest.

## Data Availability

The data that support the findings of this study are available from the corresponding author upon reasonable request.
